# GENETIC SUPPORT FOR INTERVENTIONS THOUGHT TO PROMOTE HEALTHY AGING

**DOI:** 10.1093/geroni/igz038.762

**Published:** 2019-11-08

**Authors:** Nicholas J Schork, Richard Miller, Thomas Girke

**Affiliations:** 1 The Translational Genomics Research Institute (TGen), Phoenix, Arizona, United States; 2 University of Michigan, Ann Arbor, Michigan, United States; 3 University of California Riverside, Riverside, California, United States

## Abstract

We consider the human genetic targets of many proven and hypothesized pharmacologic interventions meant to promote healthy-aging and explored not only how polymorphic these targets are in the human population, but also whether these polymorphisms have been shown to impact longevity and age-related phenotypes in general. We also determined if polymorphism in the genes or proteins modulated by healthy-aging interventions have been shown to also modulate the expression levels of those genes and proteins (i.e., act as gene or protein expression quantitative trait loci – eQTLs or pQTLs, respectively) and whether they have been subjected to mediation analyses testing their potential causal relationships to aging and longevity phenotypes. We compare our results to studies of other classes of drugs used to treat specific conditions, such as hypertension.

